# Using Text Messaging Ecological Momentary Assessment to Record Changes in e-Cigarette and Combustible Cigarette Use: Pilot Randomized Clinical Trial

**DOI:** 10.2196/66709

**Published:** 2025-03-21

**Authors:** Tucker Morgan, Michelle He, Andrew Nicholson, Omar El Shahawy, Scott E Sherman, Elizabeth R Stevens

**Affiliations:** 1 Department of Population Health, Grossman School of Medicine, New York University, 180 Madison Ave, New York, NY, 10016, United States, 1 2014962102; 2 School of Global Public Health, New York University, New York, NY, United States; 3 Department of Medicine, VA New York Harbor Health System, Veterans Health Administration, New York, NY, United States

**Keywords:** smoking, electronic cigarette, smoking cessation, ecological momentary assessment, EMA, adherence, real-time data, mobile phone, cigarette, smoker, e-cigarettes, pilot randomized clinical trial, clinical trial, pilot study, adult smoker, chronic condition

## Abstract

**Background:**

Ecological momentary assessment (EMA) provides insight into the effectiveness and feasibility of smoking-related interventions.

**Objective:**

The objective of this paper was to assess adherence to an EMA protocol and compare EMA-derived responses with measures collected through multiple surveys.

**Methods:**

A subanalysis was conducted using data from a 12-week, open-label, and 2-arm pilot randomized clinical trial among adult smokers with chronic obstructive pulmonary disease, coronary artery disease, peripheral vascular disease, or asthma in the last 12 months (n=109). Participants were randomized to either electronic cigarette (EC) or nicotine replacement therapy (NRT) treatment arms. We compared EMA data collected through automated SMS text message prompts sent to participants 4 times daily for 12 weeks, including cigarettes smoked per day (CPD), craving, and satisfaction, to survey data collected at 12 weeks. Convergent validity between survey- and EMA-reported measures was evaluated using Pearson correlation and paired *t* tests. CPD was modeled using negative binomial regression. Relative rates (RRs) of reaching at least 50%, 75%, and 100% CPD reduction between two arms were calculated using both EMA and survey data.

**Results:**

The majority of participants were non-Hispanic White (63/109, 58%) and female (60/109, 55%), and had a median age of 60 (IQR 54‐65) years. Among the 109 participants, 59.6% (n=65) were consistently adherent to the EMA protocol over the 12-week period. Median weekly EMA response rate remained high over the 12-week study period even though a modest decline was observed (week 1, 97.8% and week 12, 89.4%). The mean CPD declined significantly (week 1, mean 14.2, SD 9.9 and week 12, mean 4.6, SD 6.7; *P*<.001). EMA-derived and survey-based CPD measurements were positively correlated (*r*=0.73, 95% CI 0.6-0.82) as were measures of craving (*r*=0.38, 95% CI 0.17-0.56). No significant paired difference in CPD was observed between EMA measurements and surveys. A significant effect of time on CPD EMA data (incidence rate ratio [IRR] 1-week change 0.93; *P*<.01) and survey data was found (IRR 12-week change 0.36; *P*<.01). However, the treatment effect was not significant, which aligned with the RR results. An increase in the EC consumption was observed over time in the EC arm, with 12.1% (7/58) fully switched to EC (defined as CPD=0 and EC use>0) and 20.7% (12/58) mostly switched (defined as a reduction in CPD>75% and EC use>0) in week 12.

**Conclusions:**

EMA is a suitable method to collect recall-based smoking-related data. Though results from mixed effect modeling and RR comparisons were similar using EMA or survey data, EMA provides unique advantages, namely greater granularity in the time and the capability to detect switching patterns in near real time. These findings provide the feasibility of using EMA in developing smoking cessation interventions in future tobacco harm reduction research.

## Introduction

In the United States, smoking remains the leading preventable cause of death, responsible for 480,000 deaths and US $170 billion in health care spending each year [1]. Despite considerable progress in the 20th century, in 2021, 30 million US adults were still cigarette users [2]. The benefits of smoking cessation are well-established [1]; however, there are high rates of relapse with existing smoking cessation therapies in some groups, like those with chronic obstructive pulmonary disease (COPD) [3]. Alternate smoking cessation tools, like electronic cigarettes (ECs), have been proposed as a more appealing harm reduction intervention for people who smoke [4-6].

In public health research, a primary interest is real-world behavior; however, this can be difficult to accurately assess in a laboratory or survey setting where measures, such as cigarettes smoked per day (CPD), typically rely on retrospective self-reports. Ecological momentary assessment (EMA), which is a data collection method that uses repeated sampling to collect data throughout a participant’s everyday life, allows for the reporting of events in real-world settings and is much less time-consuming compared to traditional study visits [7]; EMA can limit issues associated with recall bias, particularly when participants are asked to recall the frequency of events over a long period [8]. Furthermore, EMA is often done remotely, thus serving as a useful tool for researchers considering remote assessments for their studies.

Interval-based EMA data collection methods have been used widely to study smoking habits and factors affecting use [9,10]. EMA data can provide useful insight into the changes in cigarette use and other factors, such as craving and satisfaction, over time and with greater granularity than study visits that are typically weeks or several months apart. Switching effects from combustible cigarettes (CCs) to ECs over an extended period, which has previously been observed through more traditional means of data collection [11], may be better understood using EMA data, particularly for vulnerable smokers with COPD and other chronic conditions.

This study assesses the comparability of smoking-related EMA measurements—CPD, craving, and satisfaction—with survey assessments completed as part of a 2-arm pilot randomized clinical trial (RCT). We also assessed the association between participant characteristics and adherence to the EMA protocol, visualized changes in EC use and CPD during program participation, and quantified the percent of individuals who switched completely to ECs over time. Furthermore, we compared the results of mixed effects negative binomial regression and relative rate comparisons of CPD reduction using EMA data versus using study visit data.

## Methods

### Study Setting and Participants

Participants were recruited via emails and phone calls between October 2020 and September 2022 from the electronic medical records of the New York University Langone Health (NYULH) system, a private hospital system serving New York, New Jersey, and Connecticut with approximately 6.8 million active patients. Patients were eligible for participation if they (1) had an ambulatory *International Statistical Classification of Diseases, Tenth Revision* (*ICD-10*) diagnosis code for COPD, cardiovascular disease, or asthma in the last 12 months; (2) for COPD patients only, a COPD Assessment Test (CAT) score ≥10; (3) were aged 21 to 75 years (the legal age for purchasing ECs in New York is 21); (4) were a current CC user (≥5 packs in a lifetime, smokes ≥4 days/week); (5) smoked at least 5 CCs per day on days they smoke CCs; (6) were motivated to quit smoking (at least a 5 on a 10-point Contemplation Ladder) [12]; and (7) possessed a phone with SMS text messaging capabilities. Potential participants were excluded if they (1), for COPD patients only, had a CAT score ≥30 (representing severe COPD) [13]; (2) reported NRT or EC use in the last 14 days; (3) had a medical condition (eg, unstable angina or heart disease) precluding use of nicotine patch or gum as determined by the study physician or by their treating physician; or (4) were pregnant or breastfeeding. Individuals who were not open to using NRT or ECs were also excluded from the study. Participants using other tobacco products were not excluded from this study.

### Ethical Considerations

The NYU Langone Health Institutional Review Board (IRB) approved the study procedures (s20-00839) and written documentation of informed consent was received before starting data collection. The original informed consent allows for secondary analyses without additional consent. All participant data were deidentified prior to analysis. Participants did not receive any incentive for counseling calls but were incentivized to complete study visits and respond to the daily SMS text message smoking diary. Participants received US $10 for completion of a survey at baseline and at 6, 8, and 12 weeks, and US $20 for completion of a survey at 24 weeks. Participants could receive up to US $40 for completing a daily smoking diary via SMS text messages (US $20 if at least 60% of assessments are completed and US $40 if at least 80% are completed). In total, participants had the possibility of receiving a total of US $100 in incentives.

### Study Design

This is a subanalysis of a 2-arm, open-label pilot RCT [14] designed to evaluate the harm-reducing effects of ECs in patients with COPD, coronary artery disease (CAD), peripheral vascular disease (PVD), or asthma. The methods of the parent study are detailed fully elsewhere [14]. Briefly, study participants were recruited from two large urban health centers’ electronic health records (EHRs) and randomized in a 1:1 ratio to either standard smoking cessation care (nicotine replacement therapy [NRT] and counseling) or ECs and counseling [15]. Randomization was stratified by sex and baseline CPD (<20 vs ≥20). Participants were screened via phone or an internet-based screening tool before enrollment.

Participants completed study visits by telephone at baseline and at 1, 2, 3, and 6 months, with the intervention period lasting 3 months. At each visit, participants completed self-report survey assessments and were asked about their issued product supply and potential harm. Sociodemographic characteristics such as age, race, ethnicity, place of birth, gender, marital status, education, work status, living situation, and homelessness were measured via self-report at baseline. In addition, condition-specific assessments (CAT [16], Clinical COPD Questionnaire [CCQ] [17,18], Seattle Angina Questionnaire-7 [SAQ-7] [19], Asthma Control Test [ACT] [20,21]) were also assessed as was motivation and confidence to quit smoking, tobacco use, and craving via the Minnesota Nicotine Withdrawal Scale (MNWS) [22].

Based on EHR diagnoses, participants were characterized into the following groups: COPD, asthma, and CAD or PVD. Participants did not receive any incentive for counseling calls, but were incentivized to complete study visits and respond to the daily SMS text message smoking diary.

### EMA Protocol

In addition to study visit surveys, during the intervention period, participants were asked to record daily CC and EC use along with measures for CC craving and EC and CC satisfaction by SMS text messaging. Since the EMA protocol was embedded within the RCT, it allowed us to directly compare the message-derived outcomes with the survey assessments across intervention groups. The details of branching logic on the texting sequence participants would receive and the phrasing of each question are presented (Figure S1 in Multimedia Appendix 1). Certain responses triggered the follow-up questions about daily use, satisfaction, and craving. If the participant reported no CC or no EC use, only craving questions were triggered. Only participants in the EC arm received questions about EC use. Due to discordance between NRT use instructions (ie, 24 h patch) and the scheduled check-ins, the NRT arm was not asked about NRT use via SMS text message check-ins. This procedure employed time-based sampling with participants receiving SMS text message prompts four times per day, breaking the day into “Early, Lunch, Afternoon, Evening” periods such that approximately 4 hours had passed between check-ins.

Participants received brief instructions sent via an introductory text and response indicators in the body of SMS text message prompts but no formal training; of note, the EMA protocol was explained to participants during the baseline and consent process. Participants were given the opportunity to opt out of receiving SMS text messages before initiation of participation and during the study. Automated SMS text message prompts were sent to participants daily over the 12-week intervention.

### EMA Data Processing and EMA-Based Measures

The SMS text message data were used to determine daily and weekly summaries for the following measures: response rate, number of CCs, EC uses (EC arm only), craving, satisfaction with the last CC, and satisfaction with the last EC (EC arm only).

#### Response Rate and CPD

We tabulated a participant’s weekly response rate as the number of responses from the participant divided by the total number of response-required texts (ie, those texts soliciting a response from the participant). To obtain an estimate of CPD—one that would be comparable to the traditional CPD measure recorded at study visits at baseline and week 12—we calculated the weekly average CPD by dividing the total number of reported cigarettes smoked in a week by 7 days. Participants with weekly response rates lower than 60% were removed from the calculation to obtain a more stable estimate of CPD and reduce downward bias. When calculating CPD for week 12, corresponding to the 3-month follow-up visit, the average CPD for the final week of EMA data collection was used rather than a total for the final day alone; we believe this average better approximates what participants would report in a visit setting compared with a 1-day total.

#### EC-Specific Measures

For the EC group, we used responses to the prompt, “Since your last report, how many separate times did you use an e-cig?” to calculate weekly averages for EC use. We defined mostly switched as >75% reduction in CPD from week 1 and use of ECs. We defined switching to ECs as those reporting 0 CPD smoked and EC use>0 in a given week.

#### Satisfaction and Craving

We calculated the average craving (range of 0‐9 with higher values corresponding to higher craving) by averaging a person’s responses to the craving question (“On a scale of 0‐9, how much do you want to smoke a cigarette right now?”) within a week. Similarly, we calculated the average satisfaction with the last cigarette and satisfaction with the last EC (range of 0‐9 with higher values corresponding to greater satisfaction) by averaging a person’s responses to the satisfaction question (“On a scale of 0‐9, how satisfying was your last cigarette/e-cig?”) within a week.

### Statistical Analysis

To evaluate predictors of EMA adherence, participants were divided into two groups: (1) those with a weekly response rate >60% or more throughout the duration of the study period and (2) those with a weekly response rate <60% at any point in the study period. Descriptive statistics were calculated by group, including sociodemographic characteristics (age, gender, race and ethnicity, marital status, education, and work status) as well as condition-specific assessments, motivation, and confidence to quit smoking and tobacco use for each group. For variables not normally distributed, median and IQR were presented. Characteristics were evaluated using Wilcoxon rank sum test for continuous variables and Fisher exact test for categorical variables.

To assess the comparability between EMA measures of CPD and more traditional measurements gathered in a survey setting, we examined the Pearson correlation coefficient between CPD reported during the final week of EMA prompting (week 12) and CPD reported at the 3-month survey. We also examined the paired difference between EMA- and survey-reported CPD at 3 months using a paired *t* test. Pearson correlation coefficient was also applied to assess the convergent validity of EMA measurements for craving compared with survey-reported measures (first item of the Minnesota nicotine withdrawal scale: “Do you have a craving for cigarettes or desire to smoke?” with responses coded from 0‐4, corresponding to “Not present” [0], “Slight” [1], “Mild” [2], “Moderate” [3], and “Severe” [4]) [23]. The EMA prompt “on a scale of 0‐9, how much do you want to smoke a cigarette right now?” was used as the comparator for craving with higher numbers corresponding to greater craving.

To assess our primary outcome of change in CPD, we used a negative binomial mixed effects model, adjusting for the intervention arm, time (weeks from baseline), and a random intercept for each individual. We compared the results obtained from surveys by building a similar model, with the time variable expressed as time from baseline to week 12 visit. The negative binomial model has been shown to be more effective for count data than a Poisson model in the presence of overdispersion [24]. CPD reduction rate was evaluated by measuring the change in CPD from the baseline to week 12 from both EMA and survey-based data. To compare reduction rates by arm at week 12, we calculated the risk ratios of reduction rates of those reaching at least 50%, 75%, and 100% reduction as reported via EMA and survey. We used the same negative binomial mixed effects models to assess the satisfaction and craving scores.

For those in the EC arm, we tabulated the number and percentage of individuals each week that demonstrated evidence of switching (CPD=0 and EC use>0) as well as mostly switching (reduction in CPD >75% and EC use>0). We also summarized weekly EMA-based measures including response rates, the number and percentage of individuals with >60% response rate, average craving, average satisfaction with last CC, average satisfaction with last EC, average CPD, and average EC uses.

All analyses were conducted using Stata (version 18; Stata Corp) and R (version 2024.04.2+764; R Foundation for Statistical Computing), and all tests were 2-tailed, with a .05 significance level.

## Results

### Adherence to the EMA Protocol

**Figure 1.**
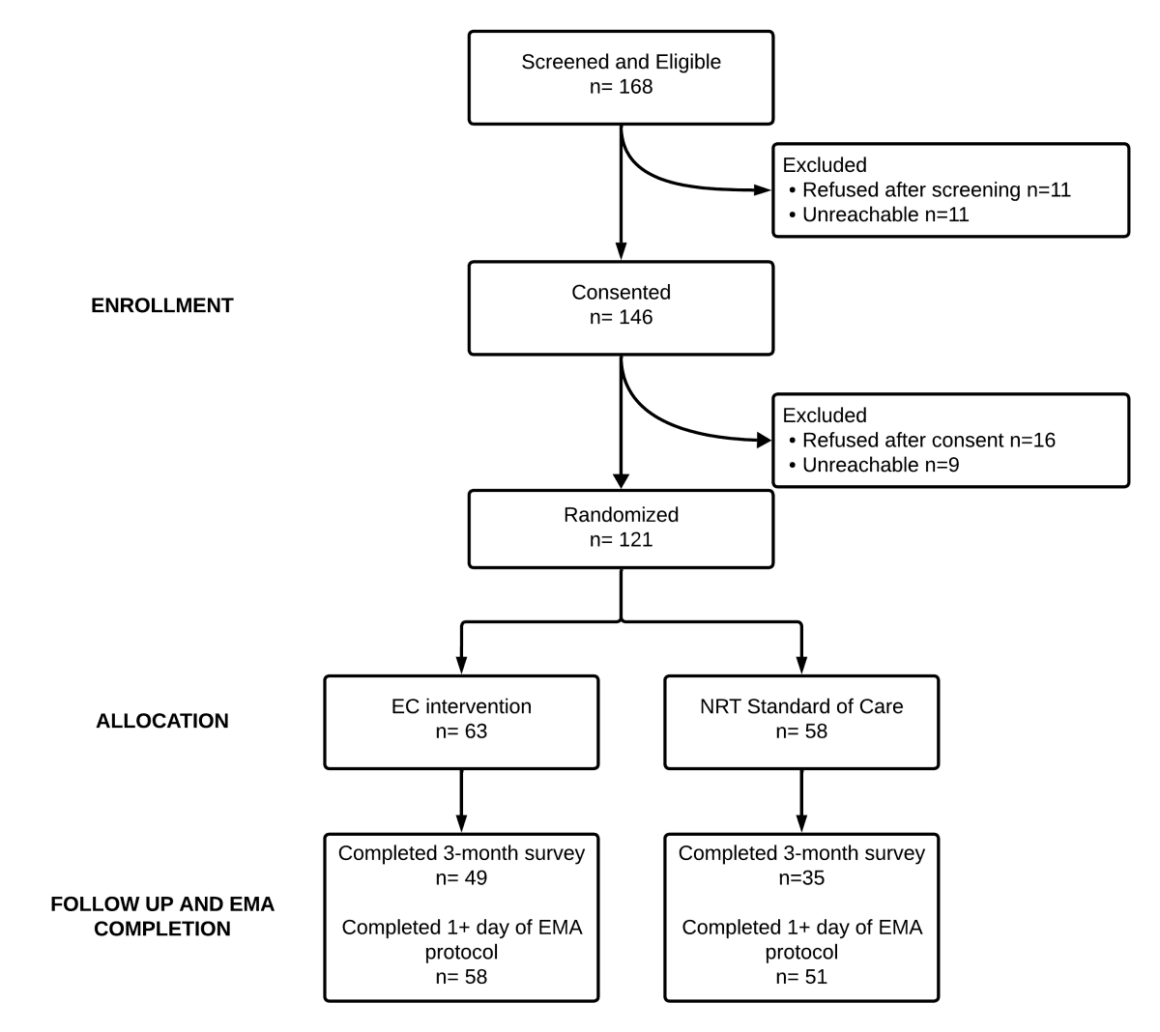
CONSORT (Consolidated Standards of Reporting Trials) flow diagram for trial recruitment and ecological momentary assessment (EMA) data analysis inclusion. EC: electronic cigarette; NRT: nicotine replacement therapy.

In total, 109 (90%) individuals of the 121 randomized trial participants took part in the EMA protocol. Among the 109, 65 (59.6%) were considered adherent to the EMA protocol with a median weekly response rate that declined slowly from 97.8% in week 1% to 89.4% in week 12. The mean and median response rates over time for all participants are plotted in Figure S2 in Multimedia Appendix 1. Of the 12 individuals with no texting data, 5 withdrew from study activities or opted out, 3 never received the study welcome text, 3 never responded to the study welcome text, and 1 died during enrollment (Figure 1).

Table 1 displays participant characteristics by adherence to EMA protocol. The median age among adherent participants was 61 (IQR 54-65) years and 58 (IQR 52-65) tears among nonadherent individuals. The sample was mostly non-Hispanic white (63/108, 57.8%), and a plurality were married or dating (52/109, 47.7%), had some college or an associate’s degree (44/109, 40.4%), and were retired or on public assistance (47/109, 43.1%). Most participants had COPD (65/109, 59.6%), followed by asthma (36/109, 33%). The median motivation to quit smoking score was 10 (IQR 8‐10), and the mean confidence to quit smoking was 6.5 (SD 2.2). At baseline, the mean number of CPD reported at the survey was 17.3 (SD 9.7), and the mean CPD from week 1 as assessed from EMA was 11.6 (SD 7.5). No significant differences were observed between the adherent and nonadherent groups.

**Table 1.** Baseline characteristics of the patients with chronic obstructive pulmonary disease (COPD), coronary artery disease (CAD), peripheral vascular disease (PVD), or asthma in the 12-week, open-label, and 2-arm pilot randomized clinical trial. Distributions and statistical metrics for the comparison of adherent and nonadherent groups to the EMA protocol are presented (n=109). All values are presented as mean (SD) or n (%) or as otherwise stated. Characteristics were evaluated using Wilcoxon rank sum test for continuous variables and Fisher exact test for categorical variables. “Adherent” participants had a weekly response rate of 60% or more throughout the duration of the study period and “Non-adherent” participants had a weekly response rate less than 60% at any point in the study period.

Characteristic	Overall	Adherent	Nonadherent	*P* value
**Participants, n (%)**	109 (100)	65 (59.6)	44 (40.4)	—^a^
**Study arm, n (%)**				.82
e-Cigarette	58 (53.2)	34 (52.3)	24 (54.5)	
NRT^b^	51 (46.8)	31 (47.7)	20 (45.5)	
**Sociodemographics**				
Age (years), median (IQR)	60.0 (54.0‐65.0)	61.0 (54.0‐65.0)	58.0 (51.5‐64.5)	.16
Sex, n (%)				.76
Male	49 (45)	30 (46.2)	19 (43.2)	
Female	60 (55)	35 (53.8)	25 (56.8)	
Race and ethnicity, n (%)				.78
Non-Hispanic White	63 (57.8)	40 (61.5)	23 (52.3)	
Non-Hispanic Black or African American	25 (22.9)	13 (20)	12 (27.3)	
Hispanic	16 (14.7)	9 (13.8)	7 (15.9)	
Non-Hispanic other^c^	5 (4.6)	3 (4.6)	2 (4.5)	
Marital status, n (%)				.67
Married or dating	52 (47.7)	28 (43.1)	24 (54.5)	
Separated, divorced, or widowed	25 (22.9)	16 (24.6)	9 (20.5)	
Single	32 (29.4)	21 (32.3)	11 (25.0)	
Educational history, n (%)				.56
Did not finish high school	11 (10.1)	6 (9.2)	5 (11.4)	
Completed high school or high school equivalency diploma (GED)	24 (22)	16 (24.6)	8 (18.2)	
Some college or associate’s degree	44 (40.4)	28 (43.1)	16 (36.4)	
Completed college or graduate school	30 (27.5)	15 (23.1)	15 (34.1)	
Work status, n (%)				.45
Full-time or part-time work	42 (38.5)	21 (32.3)	21 (47.8)	
Public assistance or retirement saving	47 (43.1)	30 (46.2)	17 (38.6)	
Unemployed or other	20 (18.3)	14 (21.5)	6 (13.6)	
**Screening diagnoses**				
Asthma	36 (33.0)	18 (27.7)	18 (40.9)	.15
CAD or PVD	24 (22.0)	13 (20.0)	11 (25.0)	.54
COPD	65 (59.6)	42 (64.6)	23 (52.3)	.20
1 or more conditions	16 (14.7)	8 (12.3)	8 (18.2)	.40
**Condition-specific assessments** ^**d**^ **, median (IQR)**				
CAT^e^	20.5 (16.0‐26.0)	19.5 (14.0‐26.0)	22.0 (16.5‐26.0)	.44
SAQ-7^f^	15.0 (13.0‐19.0)	15.0 (13.0‐19.0)	15.5 (13.5‐19.5)	.65
ACT^g^	78.8 (69.7‐87.9)	77.3 (69.7‐89.4)	78.8 (69.7‐84.8)	.79
**Dyspnea and CCQ** ^**h**^				
Dyspnea (shortness of breath) Scale				.13
Dyspnea only with strenuous exercise	18 (16.5)	13 (20.0)	5 (11.4)	
1. Dyspnea when hurrying or walking up a slight hill	27 (24.8)	19 (29.2)	8 (18.2)	
2. Walks slower than people of the same age because of dyspnea or has to stop for breath when walking at own pace	24 (22.0)	10 (15.4)	14 (31.8)	
3. Stops for breath after walking 100 yards (91m) or after a few minutes	28 (25.7)	17 (26.2)	11 (25.0)	
4. Too dyspneic to leave house or breathless when dressing	10 (9.2)	6 (9.2)	4 (9.1)	
CCQ total score, median (IQR)	2.9 (2.1‐3.9)	2.7 (2.1‐3.8)	3.2 (2.2‐4.0)	.51
CCQ symptom score, median (IQR)	3.5 (2.6‐4.5)	3.2 (2.8‐4.5)	3.8 (2.5‐4.5)	.83
CCQ functional state score, median (IQR)	2.5 (1.5‐3.5)	2.0 (1.5‐3.2)	2.6 (1.8‐3.6)	.27
CCQ mental state score, median (IQR)	2.5 (1.0‐3.5)	2.0 (1.0‐4.0)	2.5 (1.5‐3.5)	.76
**Motivation and confidence to quit smoking, median (IQR)**				
Motivation to quit (1-10)	10.0 (8.0‐10.0)	10.0 (8.0‐10.0)	9.5 (7.0‐10.0)	.72
Confidence to quit (1-10)	6.5 (2.2)	6.5 (2.2)	6.4 (2.2)	.71
**Tobacco use and risk perception, mean (SD)**				
Average number of CPD^i^	17.3 (9.7)	16.9 (9.4)	17.9 (10.2)	.60
CPD per SlickText EMA (week 1)	11.6 (7.5)	11.5 (7.1)	12.0 (8.3)	.76
**Frequency of current cigarette use, n (%)**				.09
Every day	105 (96.3)	61 (93.8)	44 (100)	
Some days	4 (3.7)	4 (6.2)	0 (0)	

a Not applicable.

b NRT: nicotine replacement therapy.

c Non-Hispanic other include Arab or Middle Eastern (n=2), not reported (n=2), and Asian (n=1).

d Condition-specific assessments restricted to those with condition: COPD: Baseline (n=65; 23 nonadherent and 42 adherent), CAD or PVD: Baseline (n=24; 13 nonadherent and 11 adherent), asthma: Baseline (n=36; 18 nonadherent and 18 adherent).

e CAT: COPD Assessment Test.

f SAQ-7: Seattle Angina Questionnaire-7.

g ACT: Asthma Control Test.

h CCQ: Clinical COPD Questionnaire.

i CPD: cigarettes smoked per day.

### Summary of EMA-Derived Measures

#### Consumption of CC and EC Over Time

Table 2 provides weekly summaries of response rates, CC and EC use, and satisfaction and craving. Using EMA, overall, the reduction of weekly average CPD was statistically significant, decreasing from 14.2 (SD 9.9) in week 1 to 4.6 (SD 6.7) in week 12 (*P*<.001). Among the EC group, the average ECs per day modestly increased from 4.3 (SD 5.5) in week 2 to 7.1 (SD 11.6) in week 10, followed by a minor decline to 5.8 (SD 9.6) in week 12.

**Table 2.** Weekly smoking-related metrics summarized from ecological momentary assessment (EMA) protocol over the 12-week study period. Satisfaction and craving values were based on a 0-9 point scale, ranging from 0 (not satisfied or no craving at all) to 9 (extremely satisfied or highest craving).

	Response rate			CC^a^ and EC^b^ use				Satisfaction and craving		
Week	Median response rate (IQR)	Mean response rate (SD)	Participants with >60% response rate, n (%)	Mean CPD^c^ (SD)	Mean EC uses per day (SD)	Participants reporting mostly switching, n (%)^d^^,e^	Participants switching to ECs^d^, n (%)	Mean Satisfaction with last CC (SD)	Mean EC satisfaction^d^ (SD)	Mean craving (SD)
1	97.8 (94.7-100.0)	95.4 (8.7)	104 (95.4)	14.2 (9.9)	—^f^	—	—	6.2 (1.6)	—	4.7 (1.7)
2	98.0 (91.1-100.0)	94.9 (8.6)	102 (93.6)	10.9 (8.9)	4.3 (5.5)	3 (5.2)	1 (1.7)	5.7 (1.8)	4.9 (2.0)	4.6 (1.7)
3	96.9 (87.5-99.2)	94.1 (10.0)	100 (91.7)	8.8 (8.1)	6.4 (7.4)	5 (8.6)	1 (1.7)	5.7 (1.9)	4.9 (2.0)	4.4 (1.7)
4	96.2 (85.8-99.1)	93.4 (10.2)	96 (88.1)	7.6 (7.6)	6.3 (7.1)	8 (13.8)	4 (6.9)	5.6 (2.0)	4.9 (2.0)	4.5 (2.0)
5	96.3 (81.6-98.5)	92.5 (11.9)	93 (85.3)	7.0 (7.5)	7.0 (9.4)	11 (19.0)	6 (10.3)	5.6 (1.9)	5.0 (1.9)	4.5 (1.8)
6	94.6 (74.9-98.5)	91.8 (12.6)	87 (79.8)	6.3 (7.5)	6.7 (7.7)	9 (15.5)	3 (5.2)	5.5 (2.0)	5.1 (1.8)	4.4 (2.0)
7	93.2 (69.4-98.6)	91.7 (13.0)	83 (76.1)	5.9 (7.2)	7.3 (9.1)	7 (12.1)	5 (8.6)	5.5 (2.0)	5.2 (1.7)	4.3 (1.8)
8	91.6 (61.5-98.3)	91.1 (13.3)	80 (73.4)	5.0 (7.3)	7.0 (10.4)	8 (13.8)	6 (10.3)	5.4 (2.0)	5.2 (1.7)	4.2 (2.0)
9	89.0 (33.3-97.6)	90.2 (14.5)	74 (67.9)	5.7 (8.7)	6.1 (9.4)	12 (20.7)	5 (8.6)	5.4 (2.0)	5.2 (1.8)	4.1 (2.2)
10	88.9 (46.5-97.9)	89.8 (13.7)	76 (69.7)	5.2 (8.1)	7.1 (11.6)	12 (20.7)	7 (12.1)	5.4 (2.1)	5.3 (1.8)	4.3 (2.2)
11	87.7 (34.9-98.0)	89.9 (13.7)	74 (67.9)	5.1 (8.2)	6.2 (10.3)	10 (17.2)	6 (10.3)	5.4 (2.1)	5.4 (1.8)	4.0 (2.2)
12	89.4 (42.5-97.8)	89.1 (15.5)	70 (64.2)	4.6 (6.7)	5.8 (9.6)	12 (20.7)	7 (12.1)	5.3 (2.2)	5.4 (1.8)	4.1 (2.2)

a CC: combustible cigarette.

b EC: e-cigarette.

c CPD: cigarettes per day.

d Summary measures refer only to the EC group (n=58).

e Mostly switching is defined as >75% reduction in CPD from week 1 and use of e-cigarettes.

f Not applicable.

#### Craving and Satisfaction Over Time

We were also able to obtain weekly estimates for craving, satisfaction with the last CC, and satisfaction with the last EC (Table 2). Weekly average craving declined modestly from 4.7 (SD 1.7) in week 1 to 4.1 (SD 2.2) in week 12. Similarly, weekly average satisfaction with the last cigarette declined from 6.2 (SD 1.6) in week 1 to 5.3 (SD 2.2) in week 12. We report weekly satisfaction and craving by arm in Table 2. No treatment effect was found in the negative binomial mixed effects models for satisfaction and craving (Tables S1 and S2 in Multimedia Appendix 1). Among EC participants, average satisfaction with the last EC rose gradually from 4.9 (SD 2.0) in week 2 to 5.4 (SD 1.8) in week 12.

### EMA Comparability With Survey Assessments

**Figure 2.**
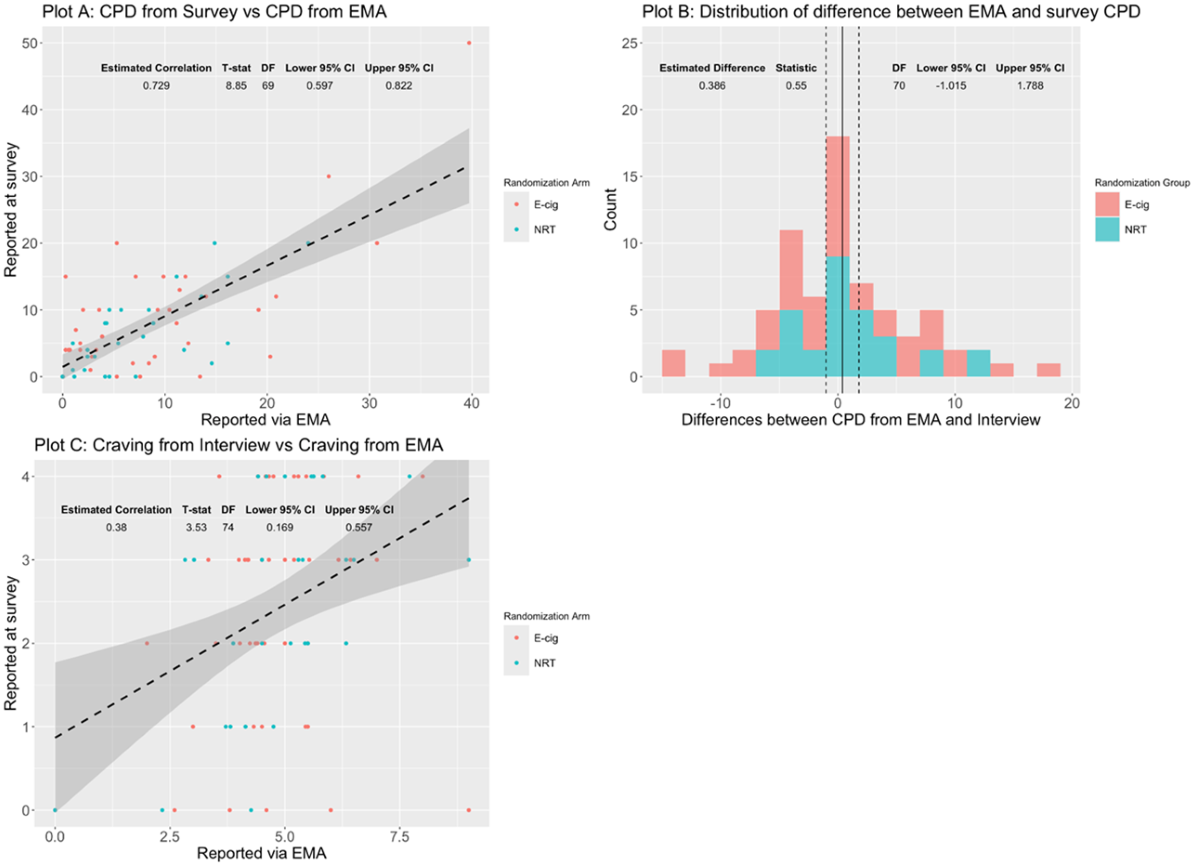
Comparison of data from both ecological momentary assessment (EMA)- and survey-based method for cigarettes smoked per day (CPD) and craving scores for combustible cigarette (CC) by treatment arms. E-cig: e-cigarette; NRT: nicotine replacement therapy.

Reported CPD estimates from EMA correlated strongly with the survey setting at 3 months (Pearson *r*=0.73; 95% CI 0.60-0.82). Of note, there was one EC participant who reported a very high consumption of combustible cigarettes (>40 CPD) via EMA and at their 3-month visit; this individual’s data was included in the current analysis as results did not differ meaningfully when excluding their data. There was no statistically significant paired difference between EMA-reported CPD and survey-reported CPD (paired difference=0.39, 95% CI −1.02 to 1.79). Craving measurements from EMA and surveys also showed a significant positive correlation (Pearson *r*=0.38, 95% CI 0.17-0.56). In Figure 2, plot A depicts a scatter plot visualizing the association between CPD use via survey and EMA at week 12. The estimated correlation (Pearson *r*) is 0.73 (95% CI 0.60-0.82). A fitted regression line and 95% CI are included. Data points are stratified by treatment arm. Plot B depicts a histogram showing the distribution of paired CPD differences between EMA and survey at week 12 for both treatment arms. The estimated mean difference is 0.39 (95% CI −1.02 to 1.79). Plot C depicts a scatter plot showing the association between craving scores for CC via survey and EMA at week 12. The estimated correlation is 0.38 (95% CI 0.17-0.56). A fitted regression line and 95% CI are included. Data points are stratified by treatment arm.

### Reduction Rate Comparisons and Negative Binomial Regression Using EMA Versus Survey Data

Tables3  4 show the comparisons between the reduction rate in CPD (50%, 75%, and 100% reduction) and negative binomial regression results using data reported through EMA and through surveys. Both treatment groups showed a significant decrease in the mean number of CPD at the end of the study period (overall; week 1: mean 14.2, SD 9.9; week 12: mean 4.6, SD 6.7) though there was no evidence that reduction in CPD was different between arms based on reduction rate. Of note, the point estimates for CPD reduction rate were all slightly higher than those computed from survey data.

The average CPD count had a mean of 8.2 (SD 8.03) and a variance of 64.5. With unequal mean and variance, our model must account for overdispersion, which the negative binomial model accomplishes. In both models, we found a significant effect of time on the average number of cigarettes per day. In the model using EMA data, we found a 7% decrease in CPD for each week (IRR for 1-week change 0.929, 95% CI 0.93-0.93), and in the model using survey data, the IRR for time across the 12-week intervention was 0.36 (95% CI 0.29-0.44). In both models, we found no statistically significant effect of randomization group on the rate of CPD; however, interestingly, the point estimates for the IRR for randomization group are in differing directions.

**Table 3.** Relative rates of reaching at least 50%, 75%, and 100% cigarettes smoked per day (CPD) reduction rates for time and treatment effects using ecological momentary assessment (EMA) and study surveys at week 12.

	EMA				Study survey			
	EC^a^ (n=58), n (%)	NRT^b^ (n=51), n (%)	RR^c^ (95% CI)	*P* value	EC (n=58), n (%)	NRT (n=51), n (%)	RR (95% CI)	*P* value
CPD reduction rate								
50% or higher	34 (58.6)	24 (47.1)	1.25 (0.87‐1.79)	.23	31 (53.4)	22 (43.1)	1.24 (0.83‐1.84)	.28
75% or higher	26 (44.8)	15 (29.4)	1.52 (0.91‐2.54)	.10	17 (29.3)	14 (27.5)	1.07 (0.59‐1.94)	.83
100%	12 (20.7)	6 (11.8)	1.76 (0.71‐4.35)	.21	10 (17.2)	8 (15.7)	1.10 (0.47‐2.57)	.83

a EC: electronic cigarette.

b NRT: nicotine replacement therapy.

c RR: relative risk.

**Table 4.** Incidence rate ratios for time and treatment effects using ecological momentary assessment (EMA) and study surveys at week 12.

	EMA			Study survey		
	IRR^a^ (95% CI)		*P* value	IRR (95% CI)		*P* value
Negative binomial regression						
Intercept	7.74 (5.34‐11.23)		<.001	15.89 (12.92‐19.54)		<.001
Time	0.929 (0.928‐0.929)		<.001	0.36 (0.29‐0.44)		<.001
Group						
NRT^b^	—^c^		—	—		—
EC^d^	0.87 (0.52‐1.45)		.60	1.11 (0.85‐1.46)		.43

a IRR: incidence rate ratio.

b NRT: nicotine replacement therapy.

c Not applicable.

d EC: electronic cigarette.

### Cigarette and e-Cigarette Use Over Time

**Figure 3.**
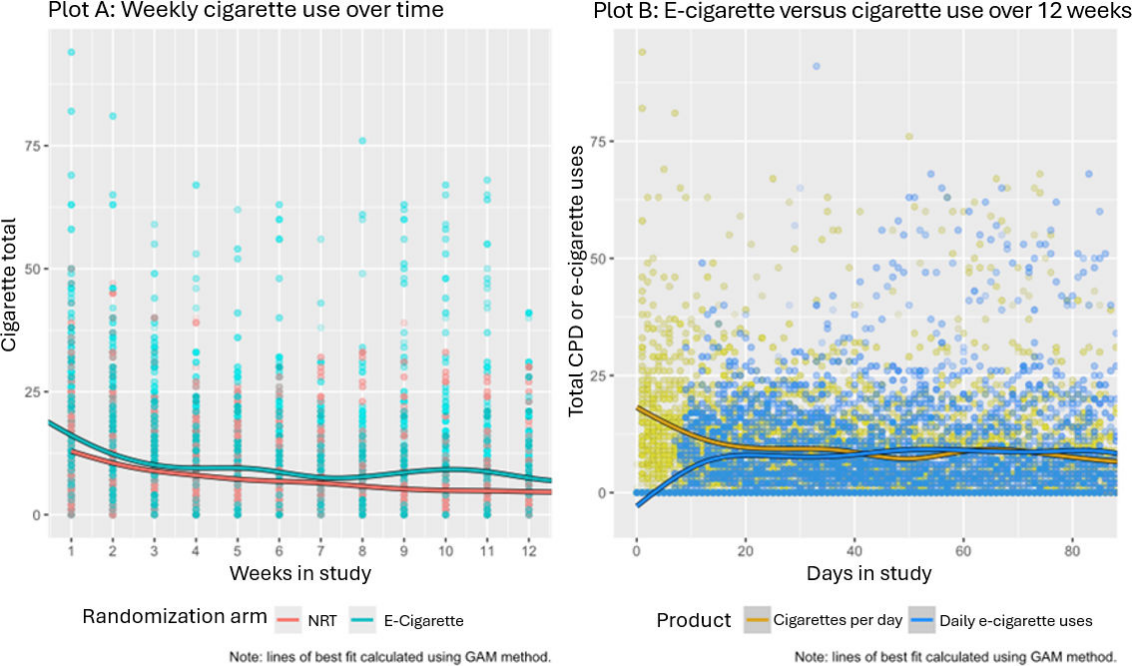
Trends in electronic cigarette (EC) and combustible cigarette (CC) use over the 12-week study period*.* Plot A depicts the change in CC by treatment arms on a weekly basis over the study period. Data points represent the total number of CC per week. A fitted line for each trial arm is included, which was fit using a generalized additive model (GAM). Plot B depicts the comparison of change in total number of daily CC and EC uses among EC arm group participants over the 12-week study period. Data points represent the total number of CC and EC uses per day. Fitted lines for CC and EC were fitted using GAM method. CPD: cigarettes smoked per day; NRT: nicotine replacement therapy.

Plot A in Figure 3 shows the reported number of CCs smoked in each week by all participants. The trend lines were fitted using a generalized additive model and are included only for visualization. This visualization aligned with the hypothesis testing of negative binomial mixed effects modeling in that we see a general downward trend in the number of cigarettes smoked for participants in each group over time, with similar trends for each group. To visualize EC use more clearly, we presented EC use in days rather than weeks.

Among those in the EC arm, EC use increased gradually and stabilized after day 20, with daily CC consumption becoming slightly lower than daily EC use. The percentage of individuals who “switched” completely from CCs to ECs in each week ranged from 1.7% (1/58) in week 2 to 12.1% (7/58) in week 12. The percentage of mostly switching increased from 5.2% (3/58) in week 2 to 20.7% (12/58) in week 12.

## Discussion

### Principal Findings

The results showed that participants in either study arm who reported greater CPD in the 3-month visit survey tended to report greater CPD in the final week of EMA prompting, and vice versa. Specifically, the EMA data indicated that EC use could lead to a reduction in CC use similar to that of NRT. These results suggest that ECs could be a potential alternative smoking cessation tool in the future. Similar to analyses using study-visit data, we found a statistically significant time effect on CPD reduction when analyzing the EMA data. Through the insights provided by EMA, patterns in dual use and switching could also be observed where participants in the EC arm appeared to consume fewer CPD over time and had a steady increase in EC use. Together, these results provide evidence that EMA is likely a suitable method to collect recall-based smoking data from participants with greater frequency and shorter recall periods compared with standard study visits.

In addition, the rigorous EMA protocol implemented in the study, with a high response rate over the 12-week intervention period, demonstrates the feasibility of assessing smoking behaviors in this manner. The relevance and validity of EMA data rely on the comparability with traditional methods of measurement (eg, surveys) and the overall adherence to the EMA protocol among participants. This study provides insight by directly comparing smoking-related measures collected through EMA and surveys. The nonsignificant paired difference found in this study contrasts with other similar studies that found large discrepancies between retrospective and EMA data [25,26]. These results indicate that an EMA protocol can achieve equal or more accurate data collection than traditional methods. Furthermore, levels of EMA adherence have been found to differ based on participant characteristics in some groups [27]. While previous studies have used EMA protocols to assess the prevalence of smoking CCs or ECs [9,26,28], as well as changing smoking patterns over time [29], this study is among the first to use a smoking EMA protocol in a population with chronic conditions, thus, demonstrating the feasibility of an SMS text messaging–based smoking EMA protocol in this population.

The visualization of CPD and EC use over time (see Figure 3) offer examples of the added information provided by EMA, giving researchers more precise data collection points. EMA can also help identify and quantify the switching (from CCs to ECs) patterns of participants closer to when switching actually occurs, which itself is an area of future research. For example, for this trial, using traditional surveys, we would only be able to assess switching at the time of surveys (at 3- and 6-month follow-ups); however, EMA allowed us to determine when and if participants did in fact switch from CC to EC within and immediately following the intervention period. If assessed in near real time, this information could even be leveraged to tailor counseling delivered to participants.

We note several limitations. In total, 109 (90%) of the 121 trial participants agreed and enrolled in SMS text messaging prompts; however, only 65 participants were adherent (EMA response rate greater than 60%) in the final week of prompting. All responses were used in the negative binomial mixed effect model, whether they responded to all prompts in a day or only a subset. This type of missingness could bias the average number of cigarettes smoked downward, particularly as fewer participants responded consistently as the study period continued. While not statistically significant, exploratory analyses seemed to suggest that employed participants may be more likely to drop off in response rates compared with retired or unemployed participants; however, further work is required to better understand effects on response rate and retention. In general, there is a risk of an EMA protocol placing too much burden upon participants leading to fatigue or burnout [30]; in turn, participant fatigue could lead to higher drop-out rates and missingness, which could bias results. Similarly, there was variance in contact intensity between the two study arms, as the NRT participants were not asked about NRT or EC use limiting the total number of questions asked. While no statistically significant difference in EMA protocol adherence was measured, differential contact between the arms could potentially bias perceived burden or engagement with the EMA protocol. Despite the missingness and the potential for participant fatigue, this EMA protocol saw continued adherence and high response rates from a majority of enrolled participants while maintaining a fairly rigorous prompt frequency. Continued adherence indicates relatively low burden of text prompts (vs through other platforms; eg, phone app) and the familiarity most participants have with texting, which could be a more acceptable and feasible method compared with lengthier surveys. Future analyses of EMA data could seek to better understand additional smoking-related measures beyond craving and satisfaction. Due to limitations of measuring experiences using numerical scales [31,32], future EMA protocols may consider free-text responses and apply tools like natural language processing to EMA data to interpret and clean patient responses thereby transforming the data into meaningful information on human behaviors and sentiments. Better implementation of set responses can help ensure researchers are receiving the information desired in workable forms and reduce the data cleaning needed. Another future area of research is determining how well EMA data can detect the real-time switching from CCs to ECs.

### Conclusion

SMS text messaging–based EMA represents a feasible and suitable method to collect recall-based smoking-related data. As compared with the survey data, the use of EMA provides unique advantages, namely greater granularity in the smoking timelines and a capability to detect switching patterns between CCs and ECs in near real time. EMA should be considered as a method for data collection when developing future tobacco harm reduction research.

## Supplementary material

10.2196/66709 Multimedia Appendix 1 Supplementary tables presenting (1) mean satisfaction with the last combustible cigarette (CC) and craving for CC over the 12-week study period stratified by treatment arms using ecological momentary assessment (EMA) data and (2) incidence rate ratios for craving and satisfaction over time and treatment effects using EMA data at week 12. Figures presenting (1) a flow diagram depicting the text messaging prompts and flow received by the patients from the electronic cigarette (EC) group (participants were asked whether they smoked a CC or used an EC since their last report and based on the responses they are directed to specific follow-up questions), and (2) trends of weekly means and medians of response rates (%) among all participants over the 12-week study period.

10.2196/66709 Checklist 1 CONSORT-EHEALTH (version 1.6.1) checklist.

## Abbreviations

ACT: Asthma Control Test
CAD: coronary artery disease
CAT: COPD Assessment Test
CC: combustible cigarette
CCD: Clinical COPD Questionnaire
COPD: chronic obstructive pulmonary disease
CPD: cigarettes smoked per day
EC: electronic cigarette
EHR: electronic health record
EMA: ecological momentary assessment
IRR: incidence rate ratio
MNWS: Minnesota Nicotine Withdrawal Scale
NRT: nicotine replacement therapy
NYULH: New York University Langone Health
PVD: peripheral vascular disease
RCT: randomized clinical trial
RR: relative rate
SAQ-7: Seattle Angina Questionnaire-7
