# Supplementary material for: Using Text Messaging Ecological Momentary Assessment to Record Changes in e-Cigarette and Combustible Cigarette Use: Pilot Randomized Clinical Trial
Source: JMIR Form Res. 2025 Mar 21;9:e66709. doi: 10.2196/66709 (PMC11951811; doi:10.2196/66709)

**Table S1. Mean satisfaction with the last CC and craving for CC over the 12-week study period stratified by treatment arms using EMA data.**

| **Week** | **Mean Satisfaction with last CC (SD)** | | **Mean craving (SD)** | |
| --- | --- | --- | --- | --- |
|  |  |  |  |  |
|  | **E-cig** | **NRT** | **E-cig** | **NRT** |
| 1 | 6.3 (1.6) | 6.1 (1.6) | 4.7 (1.8) | 4.6 (1.7) |
| 2 | 5.9 (1.7) | 5.5 (1.8) | 4.8 (1.8) | 4.4 (1.5) |
| 3 | 6.0 (1.9) | 5.4 (1.8) | 4.4 (1.8) | 4.5 (1.6) |
| 4 | 5.8 (1.8) | 5.3 (2.1) | 4.5 (2.0) | 4.4 (2.0) |
| 5 | 5.7 (1.9) | 5.5 (1.9) | 4.5 (1.7) | 4.5 (1.8) |
| 6 | 5.5 (1.9) | 5.5 (2.0) | 4.3 (2.0) | 4.5 (1.9) |
| 7 | 5.6 (2.0) | 5.4 (1.9) | 4.2 (1.9) | 4.4 (1.7) |
| 8 | 5.6 (2.1) | 5.2 (1.9) | 4.0 (2.1) | 4.4 (2.0) |
| 9 | 5.5 (2.0) | 5.4 (2.0) | 4.0 (2.2) | 4.2 (2.2) |
| 10 | 5.5 (2.0) | 5.2 (2.1) | 3.9 (2.1) | 4.6 (2.2) |
| 11 | 5.5 (2.2) | 5.2 (2.1) | 3.8 (2.1) | 4.3 (2.2) |
| 12 | 5.5 (2.1) | 5.2 (2.2) | 3.8 (2.2) | 4.6 (2.1) |

* Satisfaction and craving values were based on a 0–9-point scale, ranging from 0 (Not satisfied/No craving at all) to 9 (Extremely satisfied/highest craving)

**Table S2. Incidence rate ratios for craving and satisfaction over time and treatment effects using EMA data at week 12.**

|  | **Satisfaction** | | | **Craving** | | |
| --- | --- | --- | --- | --- | --- | --- |
|  | **IRR** | **95% CI** | **P-value** | **IRR** | **95% CI** | **P-value** |
| **Intercept** | 5.580 | 5.004, 6.221 | <0.001 | 4.244 | 3.601, 5.002 | <0.001 |
| **Weeks in progress** | 0.989 | 0.987, 0.991 | <0.001 | 0.984 | 0.982, 0.986 | <0.001 |
| **Group** |  |  |  |  |  |  |
| **NRT** | -- | -- | -- | -- | -- | -- |
| **EC** | 1.113 | 0.852,1.455 | 0.942 | 0.927 | 0.740,1.160 | 0.507 |

Abbreviations: CI, Confidence interval; EC, Electronic cigarettes; EMA, ecological momentary assessment; IRR, incidence rate ratio, NRT, Nicotine Replacement Therapy.

**Figure S1. Flow diagram depicting the text messaging prompts and flow received by the patients from EC group. Participants were asked whether they smoked a CC and/or used an E-cig since their last report and based on the responses they are directed to specific follow-up questions.**


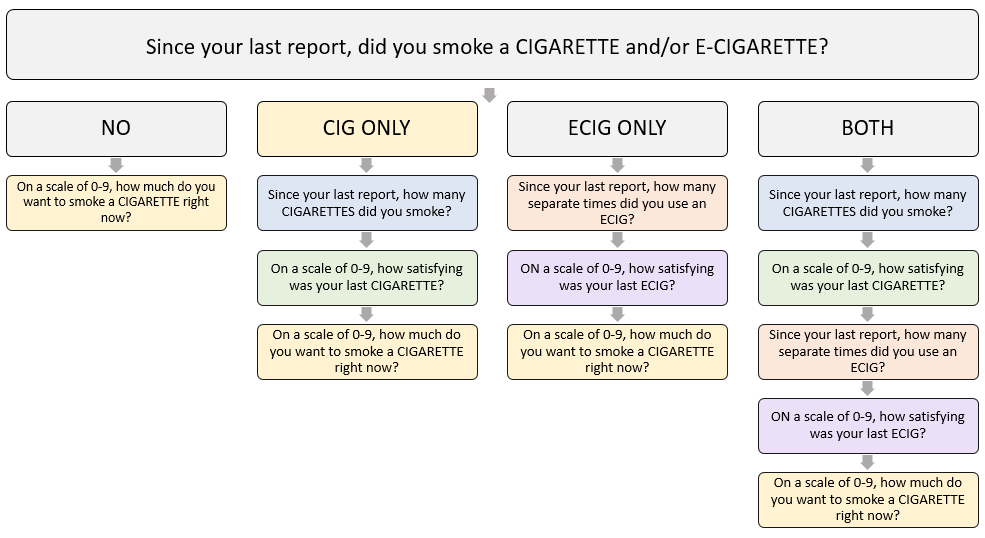


**Figure S2. Trends of weekly means and medians of response rates (%) among all participants over the 12-week study period.**


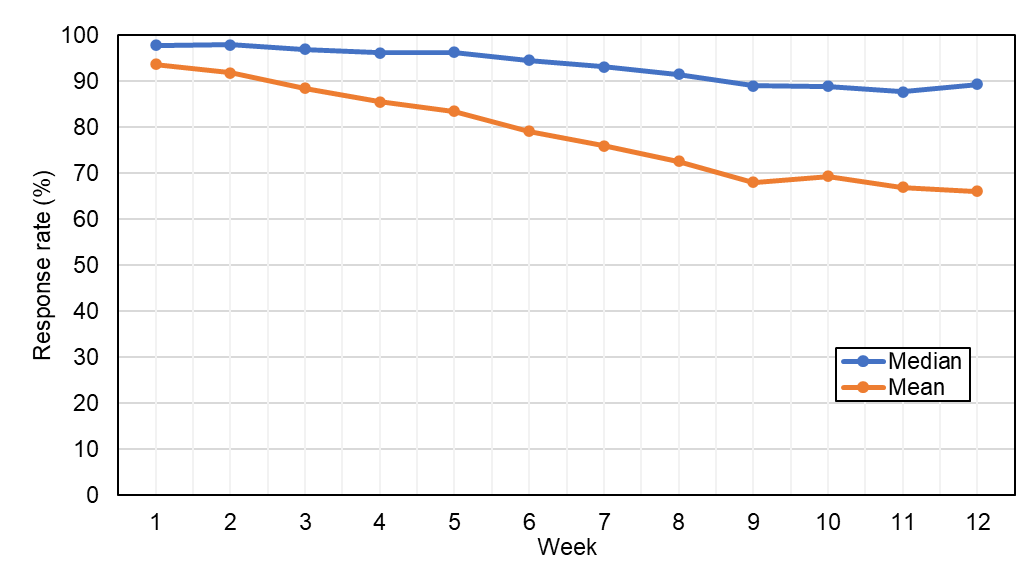

Supplement: Multimedia Appendix 1 [file formative-v9-e66709-s001.docx]
